# Theoretical Suggestions for the Improvement of Practical Surgery

**Published:** 1811-02

**Authors:** 


### *Theoretical Suggestions for the Improvement of Practical Surgery.*

(Phil. Magazine.)

1st. In that part of the operation of amputation when the bone is to be sawed through, it appears that a steady support to the bone would materially facilitate and secure the correct action of the saw: In the present mode, when the only means of steadying the bone and resistance to the action of the saw is made by the grasp and manual force of frequently agitated assistants, the difficulty of dividing the bone, without splintering and ruggedness, is very considerable. Might not a perpendicular prop from the floor, with a semicircular hollow to receive the bone, be of great effect in rendering it steady? When a retractor is used, might not such prop form part of that instrument? Carpenters, when they saw timber, always take care to make it steady previous to the application of the saw; why should not the same mode be used when sawing the bones of the arm or leg? The soft parts could not be injured by such a method; as by the present mode of amputation, by double incision, a considerable length of bone is bared before the saw is used, and why might not the proposed support be applied to that part?

2d. In the operation of trepaning the skull, when the scalp is sufficiently removed, it is essential to remove just so much of the pericranium and no more, as the head of the trephine will include; because the cranium, when denuded of its pericranium, will, like other bones denuded of their periosteum, grow carious. This part of the operation is now generally performed by an instrument called a raspatory, or by scraping the skull with a small scalpel. Would not this be performed much more complete by having a head adjusted to the trephine handle, precisely the dimensions of the serrated head, and which head would have a circular cutting edge, with a species of concave plane or scraper within it? One turn of such an instrument, which any person could easily contrive, but which is difficult to describe by words, would completely remove the exact portion of pericranium and no more.

3d. Were surgeons to make themselves acquainted with the implements used in different mechanical professions, it is possible sible some valuable additions might be made to the present instruments of surgery. They should likewise observe with a professional eye, the various mechanical improvements which are daily taking place. Might not the circular saw be introduced in some operations? Small circular saws, cutters, or wheels with toothed edges of different sizes and thickness, might perhaps be used with effect in insulating and removing the depressed angular pieces of bone which occur in fractures of the skull. When the trephine is admissible, a circular cutter applied to the edge of the fracture might, if used with proper precaution, cut away the bone with safety, and make a space sufficient to admit the elevator. Such a cutter might be turned by the hand, as great velocity would be dangerous. A method could easily be contrived to apply such an instrument with the requisite steadiness to the part.

4th. The centre point of the trephine necessarily protrudes beyond its teeth; in consequence of which, when the inexperienced operator neglects too long to remove it, the most serious effects are sure to follow. Might not this be easily prevented by having a shoulder, as mechanics term it, to surround the point, just so far down from the extremity of the point, as to permit the saw to fix itself, and no more?

5th. Would not a contrivance be useful, in trepanning the skull, to fix the head in the most favourable posture?

6th. The best shape for the points of one description of piercing instruments has never yet been exactly ascertained; and it is certainly a question of considerable importance. Those piercing instruments are meant, where breadth is requisite immediately on insertion; for, as to common needles, and other small instruments merely for piercing, it is evident the more acute their points are made, the better. In some instruments, however, where a point is merely necessary for their insertion, when that point is much prolonged beyond the *efficient* part of the instrument, it becomes injurious: What point will suit such an instrument best? Is it well ascertained that the drill or shear point is the most advantageous? If the point formed an acute angle, sloped to one side, would it not answer as well? What is the proper angle for such a point? Mechanics pierce brass, copper, and steel with drills of different shapes: May not there be an appropriate point for piercing animal membranes? The French discovered by experiments (fatal experiments!) that the descending blade of the guillotine cut best when sloped to a certain angle. However confident may be our opinion, experiment should always be had recourse to, when possible; and in satisfying the present doubts, it is very possible. An apparatus might easily be contrived for the purpose. All that is requisite is, a piece of brass, about four inches long and two inches square, supported by two or more feet, and perforated longitudinally, to admit a thick steel pin, which pin is to be fitted to the perforation in the brass, so as to move very freely, and to exceed the brass about two inches or more in length. On the upper end of this pin, a small flat piece of brass should be fixed, capable of holding weights; and in the lower end, a hole made, into which the different shaped points on which we are desirous to make experiments are to be fixed, similarly to the shifting feet of common compasses. Immediately below this pin should be placed a species of drum, consisting of a small box, with bladder or other elastic substance stretched over it to imitate the animal tunics. Now it is evident, that by placing different weights on the upper end of the pin, until the point experimenting on pierce the stretched bladder, may be exactly appreciated the comparative advantage of the different kinds of point.

7th. The operation of couching frequently fails, from the cataract or opake crystalline lens being of a soft consistency; which the couching needle, instead of depressing, passes through and divides. If the broad part, which is frequently used in depression, were made concave so as to fit the convex edge of the lens, might it not in some degree remedy this evil?

8th. Considering that the majority of calculi in the bladder are more or less spherical, it appears that the forceps now used in lithotomy is not of the most advantageous construction. By each side of the beak of the usual forceps being concave, with an oval hole at the bottom of the concavity, similar to the forceps once used in extracting polypi, the edges of which hole, and the sides of the concavity, might have teeth, Would it not be more likely to lay hold of even an irregularly spherical calculus than the forceps at present used, with toothed beaks and flat sides? and which seems better contrived for crushing a soft calculus to pieces, than for holding it fast and withdrawing it whole.

9th. Another kind of forceps for extracting the stone may be suggested. Suppose a forceps with the beaks formed of two narrow elliptical rims, jointed so as in some degree, by the pressure upon a calculus, to conform themselves to its size. To the edges of these rims attach a piece of linen or leather, forming to each beak a small purse or sack. When these forceps should be closed upon a calculus in the least spherical, the steel rims would extend to let it pass, and it would then be completely surrounded. The advantages of these forceps would be, that the calculus could could not escape; and the bulk to be withdrawn through the wound, would be very little more than the exact bulk of the calculus.

